# Sweet Basil Raw Material Functionality as a Framework for Industrial Pesto Quality: A Field-to-Fork Perspective

**DOI:** 10.3390/foods15152760

**Published:** 2026-08-06

**Authors:** Giulia Tapparo, Giorgia Botta, Andrea Caratti, Giorgio Felizzato, Erica Liberto, Marta Bertolino

**Affiliations:** 1 Department of Drug Science and Technology, University of Turin, Via Giuria 9, 10124 Turin, Italyandrea.caratti@unito.it (A.C.);; 2 Department of Agricultural, Forestry, and Food Sciences (DISAFA), University of Turin, Largo Paolo Braccini 2, 10095 Grugliasco, Turin, Italy; marta.bertolino@unito.it

**Keywords:** plant-based food systems, *Ocimum basilicum* L., functional properties, food processing, quality-by-design, functional properties, value chain

## Abstract

Sweet basil (*Ocimum basilicum* L.), the defining ingredient of “pesto alla Genovese”—a traditional uncooked Ligurian sauce prepared by blending fresh basil leaves with olive oil, pine nuts, garlic, salt, and aged cheeses- has become an increasingly important industrial raw material, yet current knowledge remains fragmented across agronomy, postharvest physiology, and food processing. This review critically proposes “raw material functionality” as an integrative framework describing the measurable ability of basil to retain the physicochemical, sensory, and technological properties required for high-quality pesto production throughout processing and storage. Evidence from the literature is critically analyzed to connect preharvest factors, postharvest handling, and processing technologies with key functional attributes, including pigment stability, phenolic content, volatile composition, enzymatic activity, microbial quality and oxidative stability. The review highlights how agronomic and technological variables jointly determine color retention, aroma preservation, shelf-life, and product consistency, while identifying major knowledge gaps in linking basil composition with industrial performance. Finally future perspectives are discussed, emphasizing predictive, quality-by-design approaches that integrate compositional markers with processing behavior and shelf-life outcomes to support more consistent and sustainable industrial pesto production.

## 1. Introduction

*Ocimum basilicum* L., commonly known as sweet basil, is one of the most representative aromatic herbs of the Mediterranean region and constitutes a fundamental component of the Mediterranean diet due to its distinctive aroma, rich bioactive phytochemical profile and long-standing culinary and medicinal use [1]. While indigenous to Asia, basil cultivation has been adopted worldwide, with the Mediterranean Basin, especially Italy, representing a center of both traditional use and commercial production [2]. In addition to its role as a fresh culinary herb, basil leaves are a source of polyphenols, phenolic acids, and flavonoids that contribute to both sensory properties and potential health benefits when consumed as part of dietary patterns associated with reduced oxidative stress and chronic disease risk [3].

Within the Mediterranean gastronomic repertoire, Pesto alla genovese stands out as an iconic sauce that exemplifies the integration of basil into traditional dishes, pairing aromatic leaves with extra virgin olive oil, pine nuts, garlic, and ripened cheese. The cultural prominence of this preparation has translated into significant industrial relevance, with jarred and ready-to-use pesto sauce widely marketed both within Italy and internationally. The global basil and pesto markets have expanded in recent years, driven by consumer demand for convenience, flavour diversity, and perceived health benefits, making pesto a valuable export commodity and a catalyst for economic activity across agriculture, food processing, and retail sectors [4].

Despite the growing body of literature on *O. basilicum*, most existing studies and reviews remain fragmented across disciplines, focusing primarily on agronomic practices, phytochemical characterization, or pharmacological properties in isolation [2,5]. Although several studies have investigated the effects of genotype, environmental conditions, and cultivation practices on basil composition, much less attention has been devoted to understanding how these factors influence technological performance during industrial pesto production [5,6]. Importantly, basil quality should be interpreted differently according to its final use [2,5,6,7,8]. Fresh-market basil is primarily evaluated according to visual appearance, leaf tenderness, freshness, and immediate aroma perception. In contrast, basil intended for industrial pesto production must be assessed according to its ability to withstand grinding, emulsification, thermal stabilization, and storage while preserving color, aroma, oxidative stability, and microbiological quality. Table 1 reports the differential quality parameters for fresh and processed basil. Therefore, fresh-market quality does not necessarily correspond to industrial processing suitability, highlighting the need for quality metrics specifically designed for industrial applications. Current research still largely investigates agronomic, postharvest, and technological factors independently, limiting the development of predictive approaches capable of linking basil composition with industrial performance and product shelf-life.

This perspective is consistent with recent trends in food science, where increasing attention is being paid to the concept of “raw material functionality” in plant-based systems, referring to the capacity of biological matrices to maintain structural, chemical, and sensory integrity throughout processing and storage [8]. Table 2 presents the key components of raw material functionality, together with their measurable indicators and relevance to industrial pesto production. Applying this concept to basil enables the establishment of direct links between agronomic practices, phytochemical composition, and industrial performance in pesto production. In this review, raw material functionality is defined as the measurable ability of basil to preserve the physicochemical, microbiological, sensory, and technological attributes required to ensure efficient industrial processing and consistent pesto quality. This functionality includes color stability, aroma retention, antioxidant capacity, enzymatic behavior, emulsion performance, microbial quality, and their combined contribution to product shelf-life.

**Figure 1:**
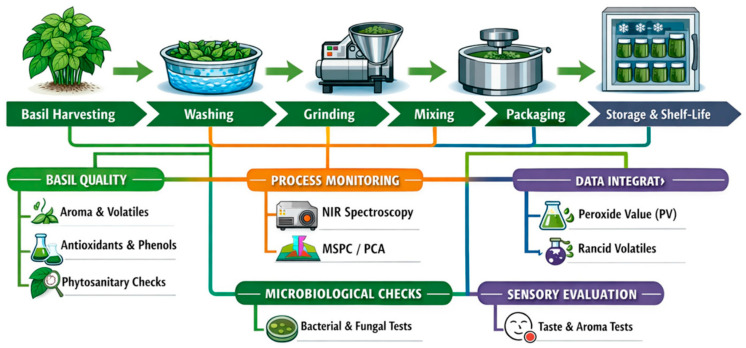
Field-to-fork conceptual framework linking agronomic determinants to technological functionality and shelf-life performance in industrial pesto.

Within the context of industrial pesto, this concept allows agronomic and postharvest variables to be directly linked to technological outcomes such as pigment degradation kinetics, lipid oxidation, microbial safety margins, and sensory shelf-life. Figure 1 reports a view of the field-to-fork conceptual framework linking agronomic determinants to technological functionality and shelf-life performance in industrial pesto. The framework highlights how pre-harvest interventions shape basil raw material functionality, which in turn influences processing behavior, quality retention, oxidative and microbiological stability, and ultimately the sensory and shelf-life performance of pesto products.

### Review Methodology

This work was conducted as a classical narrative review because the topic spans. The literature search was performed on ScienceDirect and Pubmed using keywords and combinations of terms related to *Ocimum basilicum* L., “Basilico Genovese”, basil cultivation, agronomic practices, postharvest handling, basil composition, pesto processing, quality, and shelf-life. Recent peer-reviewed studies (predominantly published between 2019 and 2025) were primarily considered, while relevant review articles and regulatory documents were included to provide historical context and production standards. The available evidence was critically analysed and integrated following a field-to-fork perspective to identify the relationships between agronomic practices, raw material functionality, technological performance, and the quality and shelf-life of industrial pesto.

The aim of this review is to provide an integrated analysis of the basil–pesto value chain and to propose raw material functionality as a conceptual framework linking basil composition with processing performance and final product quality. By contextualising current evidence within industrial challenges, this work highlights knowledge gaps and future research priorities to support more consistent and sustainable pesto production.

## 2. Sweet Basil Production Worldwide: Economic Importance and Cultivation Systems

Sweet basil (*Ocimum basilicum* L.) is one of the most economically important aromatic and medicinal plants of the family Lamiaceae and is cultivated worldwide for culinary, pharmaceutical, cosmetic, and ornamental purposes. Originally native to tropical regions of Asia and Africa, sweet basil is now extensively cultivated across temperate and subtropical regions owing to its high adaptability to different climatic conditions and production systems [9,10,11,12]. Global commercial production is concentrated in Mediterranean countries, Asia, North Africa, and North America, with Italy, India, Egypt, France, Greece, Hungary, Morocco, Israel, Indonesia, the United States, and Mexico representing some of the major producers for both fresh herbs and essential oil production. India is considered one of the world’s leading producers of basil essential oil, accounting for more than half of the global cultivation area dedicated to essential oil production, whereas Mediterranean countries dominate the fresh-market and processing sectors [12,13,14]. The global basil market was estimated at approximately USD 57 million in 2020 and is projected to exceed USD 62 million by 2026, reflecting the increasing commercial importance of the crop in both developed and emerging markets [15]. Sweet basil has become an economically strategic crop because of its short production cycle, multiple annual harvests, relatively high market value, and adaptability to open-field, greenhouse, and soilless cultivation systems [5,10]. Its versatility also makes basil suitable for sustainable cropping systems, including intercropping with cereals and legumes, improving land-use efficiency and farm profitability while diversifying agricultural production [11]. Being a key ingredient in pesto sauce, in Italy, basil production has a strong cultural and economic relevance. In specific areas such as Liguria region, basil represents a specialty protected by a designation of origin, conferring high added value to the crop. Indeed, ‘Basilico Genovese’ is recognised by the European Union (EU) as a Protected Designation of Origin (PDO) product under EU Regulation No. 611/2010. However, in this region, the Riviera Ligure is the leading production area, where ‘Genovese Gigante’ is the traditional and prevalent cultivar, accounting for approximately 90% of the total basil-growing area. The cultivar is particularly well suited for fresh consumption and is also widely used for the industrial production of the traditional pesto [4,15,16]. The increasing demand for pesto has led to a significant expansion of basil cultivation [10,15]. Indeed, in recent years, the traditional cultivation area of sweet basil has extended beyond the Riviera Ligure to include the southern provinces of the neighbouring Piedmont region. In recent years, the traditional cultivation area of sweet basil has extended beyond the Riviera Ligure to include the southern provinces of the neighbouring Piedmont region. In both Liguria and Piedmont, basil is predominantly cultivated under greenhouse conditions, where nighttime temperature decreases, and dew formation can favour disease outbreaks [15]. Currently, approximately 28,000 ha are dedicated to basil production in Italy, with about 70% located in northern regions, 10% in central areas, and 20% in southern regions. Around 1000 ha are cultivated in open fields, with the Emilia-Romagna region accounting for approximately 30–40% of this area [14].

Basil intended for industrial processing is primarily grown in open fields at sowing densities ranging from 15 to 30 kg of seed per hectare, allowing at least 4–5 harvests per year. Its cultivation is particularly sensitive to climate conditions: the basil growing cycle in open fields requires a mild season, making cultivation possible from May to September in temperate zones. Therefore, growing a healthy crop managed with proper cropping systems and suitable inputs is crucial. Production is typically carried out on large-scale farms equipped with overhead sprinkler or drip irrigation systems and mechanical harvesting technologies. In contrast, basil for fresh consumption is mainly cultivated under protected conditions (plastic tunnels or greenhouses), where temperature and light can be partially controlled and preventive measures against pests and diseases can be implemented [14,17]. In recent years, soilless cultivation, particularly hydroponic systems, has become increasingly widespread due to shorter production cycles, more frequent harvests, and improved leaf tenderness. Moreover, beyond these production advantages, hydroponic cultivation of basil and coriander has been shown to yield pesto with superior quality attributes and shelf-life compared with soil-grown counterparts [10]. In addition, soilless systems enhance sanitary quality due to the absence of soil residues and a reduced risk of microbial contamination or heavy metal accumulation [17,18].

## 3. Cultivar and Preharvest Factors Shaping Basil Quality

### 3.1. Cultivars Used for Pesto Sauce Production

Within *Ocimum basilicum*, a large number of cultivars and botanical varieties have been identified, showing high morphological, chemical, and aromatic variability [3]. Among them, the sweet basil “Genovese” is one of the most renowned, traditionally cultivated in the Ligurian basin (Italy) for the production of fresh leaves used in pesto, a product certified as Protected Designation of Origin (PDO) [2,19]. According to the production specifications for Basilico Genovese DOP, seeds must derive from *O. basilicum* native accessions, ensuring genetic continuity and the preservation of the cultivar’s typical traits [20]. Under the Ligurian pedoclimatic conditions and using traditional cultivation techniques, these plants fully express their distinctive features. Morphological traits include a medium-high to tall growth habit, an expanded or cylindrical canopy, elliptical leaves with flat or slightly convex blades, slightly dentate margins, and surfaces that are minimally or not at all blistered. From an organoleptic perspective, Genovese Basil PDO is characterized by a strong floral note due to linalool and the absence of estragole, which would otherwise confer a mint/anise aroma considered undesirable [6,20,21]. These qualities make the cultivar particularly suitable for the preparation of traditional Pesto Genovese. However, production is limited to a small geographic area and in limited quantities, making it unsuitable for large-scale industrial pesto production [21].

To meet industrial demand, breeding programs have produced new *O. basilicum* cultivars, selected based on aroma, flavor, leaf morphology, plant height, internode number as well as yield, adaptability to open-field cultivation, regrowth capacity, and flowering control [22,23,24,25].

According to De Masi et al. [26], the “Genovese” cultivar, characterized by high linalool (46.7–53.9%) and eugenol (9.7–20.9%) content, is primarily used for “Pesto Genovese” production. Other cultivars, such as “Red Dark Opal” and “Fine Verde,” display essential oil compositions similar to Genovese accessions, while “Lettuce Leaf” is more suited for food flavoring. Notably, phenotypic variations may occur within the same Genovese cultivar depending on the seed supplier. Seeds from P.D.F., Scafati (SA), lead to plants that are generally shorter, with lighter, broader, and more oval leaves, whereas those from Sementi Sommi, Parma (PR), tend to be taller, with darker, lanceolate, and medium-sized leaves [26,27]. If the U.S. market for fresh and dried aromatic herbs appears to be dominated by the “Genovese”, “Italian large leaf”, “ Mammoth”, “Napoletano” and “Sweet” cultivars, reported that in Italy, at least 12 cultivars are currently evaluated for industrial pesto production among which “Italiano Classico” is the most widely used commercially [28,29]. This cultivar is characterized by higher eucalyptol and eugenol content and lower linalool levels compared to cultivars such as “Aroma 2” or “Eleonora”, the latter being among the first *Peronospora*-resistant cultivars to be commercialized [30].

*O. basilicum* is susceptible to several pathogens, including *Fusarium* spp. and *Peronospora* spp., the latter being the causal agent of downy mildew, the most widespread disease in sweet basil. Infection leads to leaf yellowing, browning, and eventual defoliation, causing significant yield losses and marketability issues. The development of downy mildew-resistant cultivars represents the most sustainable control strategy, albeit limited for Genovese PDO basil. Nevertheless, the high genetic variability of *P. belbahrii* necessitates continuous updates in integrated pest management and breeding programs. Thanks to investments by seed companies, pathogen-tolerant cultivars that maintain the organoleptic traits of the Genovese type have been developed, including “Zeus”, “Diamante”, “Paoletto”, “Garibaldi”, “Gentile”, “Gemini”, and “Basiglio”. Continuous field monitoring of these cultivars remains essential, as the pathogen can evolve into new races, as observed in New Jersey [15,31].

Phytochemical characterization of basil has highlighted the central role of phenolic compounds, volatile chemotypes, and pigments. Phenolics such as rosmarinic and caffeic acids are strongly influenced by genotype, environmental stress, and seasonal variation, leading to significant fluctuations in antioxidant capacity [4,32]. However, although their antioxidant properties are well established, their effectiveness in complex systems such as pesto remains poorly quantified. In emulsified matrices rich in lipids, oxidation is governed by interfacial mechanisms involving oxygen and pro-oxidant metals, meaning that phenolic activity depends not only on concentration but also on molecular interactions and partitioning behavior [33,34]. Chemotyping further contributes to basil variability, with distinct profiles dominated by compounds such as linalool, eugenol, or estragole [35]. These profiles are highly sensitive to environmental conditions, including water stress and storage, which can significantly alter aroma intensity and stability. Despite this knowledge, no standardized chemotype criteria exist for industrial pesto production, and the relationship between volatile composition and aroma persistence in processed products remains largely unexplored. Color, primarily determined by chlorophyll content, represents another critical attribute linking raw material quality to consumer acceptance. While agronomic factors such as nitrogen supply and light intensity enhance chlorophyll accumulation [5], pigment stability during processing is limited. Chlorophyll degradation under acidic and thermal conditions leads to pheophytin formation and color loss [36]. Importantly, the relationship between initial chlorophyll content and color stability (e.g., ΔE evolution during storage) remains poorly defined, as degradation kinetics depend more strongly on processing conditions than on initial pigment levels. Currently, no universally accepted thresholds exist for defining the minimum or optimal volatile composition for pesto production, resulting in variability in sensory quality across batches. This lack of standardization highlights the need for industrial quality benchmarks based on functional parameters rather than solely botanical classification. In addition, the direct relationship between basil phytochemical composition and pesto stability remains insufficiently explored. Phenolic compounds, particularly rosmarinic acid, are known to exhibit strong antioxidant activity and may contribute to the inhibition of lipid oxidation in oil-rich systems [5,37]. However, limited studies have quantitatively linked phenolic concentration in basil leaves to oxidative stability, color retention (ΔE), or chlorophyll degradation in pesto matrices. Similarly, the role of phenolics in modulating chlorophyll stability and delaying the formation of pheophytins during thermal processing has not been fully elucidated. Establishing these relationships would enable the identification of compositional markers predictive of processing performance, representing a key step toward a functionality-driven classification of basil raw materials.

### 3.2. Pre-Harvest Factors Shaping Basil Quality

The quality of basil intended for industrial pesto production is strongly influenced by agronomic management, and environmental conditions, which modulate leaf color intensity, aromatic profile, phenolic composition and nitrate accumulation.

#### 3.2.1. Geographical and Climatic Conditions

*O. basilicum* is cultivated across a wide range of agro-ecological environments. Although it prefers warm climates, it adapts to conditions ranging from tropical regions to cooler (6–24 °C) [38]. Geographical location and edaphoclimatic factors are major determinants of essential oil yield and chemical composition [25]. Substantial chemotypic variability has been reported worldwide, with marked regional differences in dominant volatile compounds. For example, European varieties are commonly characterized by linalool-rich profiles, whereas basil grown in Asia and Pacific regions may show higher levels of camphor, limonene, β-selinene, or methyl cinnamate [39]. Recent evidence further confirms that even within the same cultivar, local environmental conditions significantly modulate secondary metabolite profiles. López-Hernández et al. [12] demonstrated site-dependent variation in the essential oil composition of the “Nufar” cultivar in Colombia, reinforcing the role of edaphoclimatic drivers in shaping basil chemotypes.

From an industrial perspective, site-driven chemotype shifts should be interpreted in terms of processing suitability: changes in dominant terpenes and phenylpropanoids can alter the headspace aroma of pesto and its sensory shelf-life trajectory, underscoring the need to couple terroir studies with GC–MS fingerprinting of finished products [28,40].

#### 3.2.2. Agro-Environmental Factors Shaping Basil Quality

Agro-environmental factors play a central role in shaping basil quality, influencing biomass production, phytochemical composition, and ultimately the functional performance of the raw material in industrial applications. A substantial body of literature has demonstrated that environmental conditions such as water availability, seasonal variation, and climatic factors significantly modulate the color and both volatile/phenolic profiles in *O. basilicum* [5,35]. Indeed *O. basilicum* quality for pesto production is defined by multiple biochemical and sensory attributes that influence both the nutritional value and consumer acceptance of the final product. Basil used for pesto is prized for its bright green leaf color, which correlates with high chlorophyll and carotenoid content and is considered a key indicator of fresh product quality [13]. The aromatic profile of basil, driven by volatile compounds such as linalool, eugenol, and other terpenes, determines the characteristic herbaceous and sweet sensory notes essential to traditional pesto flavor. Total phenolic content, including phenolic acids like rosmarinic and caffeic acid, contributes significantly to basil’s antioxidant capacity and is positively associated with nutraceutical quality, shelf-life stability, and perceived freshness. At the same time, nitrate accumulation in leafy tissues is monitored as a critical anti-nutritional factor, since high nitrate levels can pose food safety concerns despite being influenced by genotype and harvest practices.

These findings indicate that basil phytochemical profiles are inherently dynamic, complicating the standardization of raw material quality for industrial processing.

#### 3.2.3. Water Needs

Watering method, frequency, quality, and overall irrigation strategies strongly affect biomass production, growth performance, and final yield [41]. Excessive irrigation or heavy rainfall can promote waterlogging, impairing root oxygenation and nutrient uptake while increasing disease incidence, ultimately reducing plant growth and productivity [42]. In contrast, deficit irrigation (DI) generally leads to reduced biomass accumulation due to limited vegetative development, particularly in vigorous cultivars such as “Genovese” basil [41,43]. Water stress, in particular, has been extensively investigated as a key driver of metabolic responses [35,44]. On the one hand, DI, although reducing biomass, may enhance the total volatile concentration through stress-induced metabolic pathways; on the other hand, it may also reduce key aroma compounds such as linalool, geraniol, and eugenol, with effects that can persist during postharvest storage [35,45]. In addition, moderate abiotic stress has been associated with increased accumulation of phenolic compounds, resulting in enhanced antioxidant capacity [4,5]. Regarding postharvest quality, moderate DI (25%) does not significantly affect visual quality or chlorophyll content, whereas severe DI (50%) reduces visual quality and increases oxidative stress, despite simultaneously enhancing antioxidant capacity. Since respiration rate is closely linked to tissue deterioration and shelf-life, excessive water stress may ultimately compromise postharvest performance. Notably, irrigation-driven changes in antioxidant status and volatile retention become technologically relevant only if they effectively reduce oxidation rates and slow aroma loss once basil is dispersed in oil-rich emulsions, where interfacial oxidation is a major deterioration mechanism [34].

#### 3.2.4. Light Management

Light management is another critical determinant of basil quality for pesto processing. Light intensity affects photosynthetic performance and phenolic accumulation, whereas excessive shading may increase nitrate content and alter volatile composition [46,47]. In controlled environments, LED technology enables spectral manipulation to optimize yield and quality. Appropriate red:blue light ratios promote biomass accumulation and chlorophyll content, while blue light alone or in combination with other wavelengths, enhances phenolic biosynthesis. Moreover, short-term UV-B applications at the final growth stages can increase polyphenol concentration without severely affecting yield, representing a promising strategy to improve nutraceutical quality before processing. While controlled light strategies, including LED-based spectral manipulation, have been shown to enhance specific phytochemical traits, their relevance for industrial applications remains unclear. In particular, the extent to which light-induced increases in pigments and antioxidants translate into improved color stability and oxidative resistance in pesto has not been systematically investigated. Furthermore, pigment enrichment at harvest does not guarantee greener pesto because chlorophyll stability is ultimately governed by process parameters like pH, oxygen exposure and, particularly, thermal history which accelerate pheophytinization and browning in pasteurized products [36,40].

#### 3.2.5. Mineral Nutrition

Mineral nutrition plays a central role in determining basil quality. Nitrogen is directly associated with chlorophyll and protein synthesis, thus influencing the intense green color required by the processing industry. Adequate nitrogen supply enhances biomass production and essential oil yield; however, excessive nitrogen fertilization may lead to nitrate accumulation in leaves, greater susceptibility to stress and nutritional imbalances [5,48,49]. The adoption of slow-release fertilizers or targeted nitrate management in hydroponic systems improves nitrogen use efficiency and helps limit leaf nitrate content. Potassium is equally important, as it promotes the biosynthesis of secondary metabolites, including phenolic acids and flavonoids, thereby contributing to antioxidant capacity and sensory attributes [37,50]. Phosphorus also plays a key role by stimulating phenylalanine ammonia-lyase (PAL) activity and enhancing the accumulation of rosmarinic and caffeic acids [51].

In soilless cultivation systems, precise nutrient solution management enables fine modulation of phytochemical composition. Controlled nutrient limitation or partial nitrate substitution can significantly reduce nitrate accumulation without severely compromising yield [52,53]. Furthermore, the application of moderate abiotic stress, such as controlled salinity, has been shown to increase phenolic concentration and antioxidant activity, potentially improving oxidative stability of processed products, although with possible reductions in productivity [5,54].

This highlights a recurrent trade-off between agronomic functionalization and industrial scalability: nutritional strategies that raise phenolics may support oxidative stability, yet reduced yields and altered tissue composition can increase variability at reception and require tighter process control to achieve consistent pesto quality [5,40].

#### 3.2.6. Biofortification

Biofortification strategies may further influence nutritional and functional quality. Selenium enrichment has been associated with increased phenolic compounds and essential oil content, thus potentially improving aromatic characteristics [55]. Nevertheless, selenium concentrations must be carefully optimized to avoid phytotoxicity or yield penalties. Similarly, iodine biofortification may enhance phenolic accumulation, although excessive doses can negatively affect plant growth [56].

#### 3.2.7. Biostimulation

Biostimulant applications represent an additional tool to improve aromatic and functional quality. Seaweed extracts have been reported to increase essential oil concentration and key volatile compounds such as linalool and estragole [5,57]. Microbial biostimulants, including plant growth-promoting rhizobacteria (PGPR) and arbuscular mycorrhizal fungi (AMF), can stimulate rosmarinic acid biosynthesis and modulate essential oil composition, while simultaneously enhancing tolerance to abiotic stress [5,58,59].

The available evidence suggests that agronomic management can be regarded as a tool for tailoring basil raw material functionality. Several pre-harvest interventions have been shown to alter biomass production, antioxidant capacity, phenolic composition and aroma-related metabolites, traits that are expected to influence pesto quality and shelf-life. A synthesis of the main agronomic drivers and their potential technological implications for pesto production is reported in Table 3.

**Figure 2:**
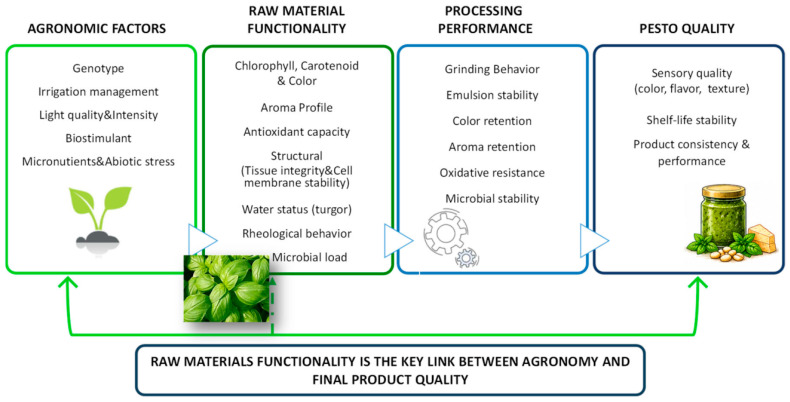
Raw material functionality concept.

Despite these advances, current agro-environmental research remains largely focused on compositional outcomes at harvest, with limited integration into processing-oriented frameworks. For example, although stress-induced increases in phenolic compounds are well documented, their effectiveness in inhibiting lipid oxidation in complex emulsified systems such as pesto, where interfacial phenomena dominate, has not been quantitatively established [60]. Moderate abiotic stress enhances phenolic accumulation; however, its translation into improved processing stability remains unproven, as most studies fail to link compositional changes to oxidation kinetics or shelf-life performance [35,45]. However, these benefits are often accompanied by reductions in biomass and overall productivity, raising questions regarding their practical applicability at an industrial scale. This highlights the need for a “controlled stress strategy,” in which stress levels are optimized to enhance phytochemical composition without compromising economic yield. Similarly, alterations in volatile composition due to environmental stress have rarely been linked to aroma persistence or sensory decay in processed products [35]. Moreover, the interaction between multiple environmental factors remains insufficiently characterized. Most studies adopt single-factor experimental designs (e.g., water stress or seasonal variation alone), whereas real cultivation systems involve simultaneous interactions between light, temperature, water, and nutrient availability. This limits the development of predictive models capable of linking agro-environmental conditions to raw material functionality and industrial performance Figure 2.

Another critical gap concerns the absence of threshold-based criteria defining optimal cultivation conditions for processing applications. While moderate stress may enhance phytochemical composition, the lack of quantitative benchmarks prevents the identification of an optimal balance between yield, compositional stability, and technological performance.

A central challenge emerging from agronomic studies is the trade-off between yield and functional quality. From a processing perspective, such strategies can be interpreted as a form of agronomic priming aimed at improving raw material functionality, particularly in terms of oxidative stability and aroma retention during pesto production. Moreover, while nitrate accumulation has been extensively studied from a nutritional and safety standpoint, its potential impact on processing stability remains poorly understood. Variations in nitrate content may influence pH buffering capacity, microbial growth dynamics, and water activity in the final product, suggesting a possible indirect role in determining pesto shelf-life. This represents an underexplored research area requiring further investigation.

Overall, agro-environmental research has generated substantial knowledge on the drivers of basil composition, yet remains largely disconnected from food processing and shelf-life studies. Bridging this gap requires an integrated approach in which agronomic variables are systematically linked to processing behavior, enabling the development of predictive models that define basil quality in terms of functional performance rather than compositional descriptors alone.

## 4. Harvest and Postharvest Factors Shaping Basil Quality

Harvest and postharvest factors are closely interconnected and jointly contribute to the final quality of basil. Harvesting operations, including timing and handling, can markedly influence the preservation of sensory and nutritional attributes, while postharvest practices further affect their stability during storage and processing. For this reason, harvest and postharvest factors are discussed together in this section as consecutive stages of the same production chain.

*Ocimum basilicum* is typically cultivated from April to September, and harvest timing is strongly influenced by ontogenetic stage [14]. Increasing evidence indicates that harvest time significantly affects both physiological status and postharvest quality. Early morning or late afternoon harvests favor carbohydrate accumulation and reduce photoinhibition, whereas midday harvest increases pigment concentration but may induce transient light stress [61]. Moreover, reduced chilling injury during storage has been observed in material collected in the early afternoon and harvesting in the early evening has been associated with extended shelf-life compared to early morning harvests [1]. Overall, diurnal timing represents a critical harvest factor influencing biochemical traits and storage performance. For processors, diurnal effects are relevant insofar as they shift enzyme activity and volatile pools at the moment of grinding, conditions that can modulate enzymatic browning and early aroma release in the semi-finished basil–oil matrix [4,40].

Immediately after harvesting, this highly perishable raw material has to be preserved against deterioration and spoilage [36].

### 4.1. Cuttings and Growth Stage

Essential oil biosynthesis in basil is closely linked to plant developmental stage. Oil production is highest in young, actively growing tissues, particularly during early leaf expansion, and tends to decrease proportionally as leaf biomass increases due to volatilization and dilution effects.

To maximize essential oil yield, both cultivar selection and optimization of successive harvests are considered strategic approaches. However, repeated cuttings may induce mechanical stress, potentially affecting qualitative and sensory attributes. Field studies on “Genovese” cultivars have shown that successive harvests can increase fresh yield and dry biomass, likely due to enhanced photosynthetic efficiency, while reducing the leaf-to-stem ratio without significantly altering dry matter content [4,60]. Collectively, plant developmental stage and harvest frequency are key determinants of yield performance and qualitative traits in basil production.

### 4.2. Storage Conditions

Chilling sensitivity is a major limitation in the postharvest handling of sweet basil, as severe chilling injury occurs at ≤5 °C, with leaf necrosis developing after only 1 day at 0 °C or 3 days at 5 °C. Shelf-life is maximized at 12–15 °C and declines sharply at 10 °C, 5 °C, and 0 °C [62,63]. Storage below 12 °C also reduces key volatile compounds and other secondary metabolites responsible for aroma, antioxidant capacity, and visual quality [63,64]. However, cooler temperatures (<15 °C) are more effective in limiting microbial growth compared to 20–22 °C [65,66]. Therefore, moderate refrigeration at 12–15 °C represents the optimal compromise to minimize chilling injury, preserve aroma, and control microbial development during storage and distribution.

Because industrial pesto relies on rapid comminution and stabilization, cold-chain decisions should be evaluated against downstream outcomes such as Polyphenol oxidase and Peroxidase (PPO/POD)-driven browning during grinding and aroma losses during pasteurization, not only against fresh-leaf appearance [40,63].

Postharvest artificial lighting may partially mitigate senescence and quality loss in sweet basil during storage. Prolonged darkness can accelerate leaf senescence in detached tissues, and storage under continuous fluorescent light (4.6 μmol m^−2^ s^−1^) at 12 °C improved aroma retention compared to dark conditions [67]. However, continuous illumination may also enhance physiological activity, chlorophyll degradation, and transpiration, potentially shortening the shelf-life [68]. In basil, short daily pulses (2 h) of low-intensity white or red light (30–37 μmol m^−2^ s^−1^) during storage at 20 °C helped maintain chlorophyll and protein content, reduce ammonium accumulation, and increase soluble sugars, indicating delayed senescence, likely mediated by phytochromes. UV-B exposure (3.60 W m^−2^ for 8 h) also showed potential to preserve secondary metabolites, although results were limited to short-term assessments [69]. Overall, controlled, low-intensity or pulsed artificial lighting appears more beneficial than continuous illumination, offering a promising strategy to delay senescence and maintain postharvest quality. Controlled atmosphere (CA) storage can extend the postharvest life of sweet basil when low O_2_ and minimal or no CO_2_ are maintained. Reducing O_2_ slows respiration, aging, and ethylene activity, and an atmosphere of 1.5% O_2_ + 0% CO_2_ (in N_2_) achieved a shelf-life of up to 45 days in micro-perforated bags, compared to 18 days in air at 20 °C [1]. However, O_2_ levels below 1% caused dark, water-soaked lesions, while elevated CO_2_ (>5%) reduced shelf-life and induced interveinal browning, with severe quality losses reported at 7.5–10% CO_2_ or higher. Basil is particularly sensitive to CO_2_ compared to other herbs [67,70,71,72]. Although 0% CO_2_ appears optimal under N_2_-based CA, moderate CO_2_ enrichment (e.g., 2.5 kPa or 5%) under ambient air may reduce decay, browning, and leaf abscission, especially when combined with 1-methylcyclopropene (1-MCP), an ethylene action inhibitor, suggesting a role of CO_2_ in modulating ethylene-mediated senescence [1]. Overall, optimal CA management for basil involves low but not anoxic O_2_ and minimal CO_2_ to avoid physiological injury while slowing metabolic deterioration. Although CA optimization remains largely leaf-centric, future work should quantify whether CA-preserved volatiles and antioxidants measurably extend pesto shelf-life or reduce oxidation markers after formulation, where oxygen management and emulsion microstructure dominate deterioration pathways [33].

### 4.3. Humidity

Maintaining a humid environment in postharvest storage helps maintain freshness and prolong shelf- life. The Rutgers New Jersey Agricultural Experiment Station recommends a high relative humidity of 90–95% for the storage of basil. This helps maintain leaf quality and slow enzymatic activity. The high humidity itself may even enable basil to be stored at lower temperatures by minimizing water loss [1,73].

### 4.4. Packaging

Packaging plays a key role in preserving the postharvest quality of sweet basil, with low-density polyethylene (LDPE) widely used due to its high gas permeability, moisture barrier properties, chemical inertness, and cost-effectiveness [73]. Perforated polyethylene (PE) liners have been shown to reduce weight loss and better maintain visual quality compared to perforated polypropylene (PP) or paper-lined cartons during storage at 10 °C followed by 18 °C. Microperforated plastic packaging without modified atmosphere packaging (MAP) performed better than non-perforated films with high CO_2_ atmospheres by minimizing water loss, leaf abscission, and decay, likely through reduced CO_2_ and ethylene accumulation [67]. However, non-perforated LDPE combined with a moderate MAP (10.5% O_2_ and 4.2% CO_2_) doubled shelf life (8 to 16 days) at 11–12 °C compared to macroperforated LDPE, suggesting that controlled gas composition within LDPE can be advantageous when CO_2_ levels are carefully managed. Further improvements may be achieved by minimizing CO_2_ accumulation [62,70,74]. In some cases, LDPE packaging has also allowed storage at lower temperatures (5 °C) while preserving biochemical attributes such as chlorophyll, phenolics, flavonoids, antioxidants, and membrane integrity, although effects on visual quality and aroma were not fully assessed [75]. Overall, optimized LDPE-based packaging, particularly when combined with carefully regulated modified atmospheres, represents an effective strategy to reduce water loss, modulate gas exchange, and extend basil shelf-life.

Packaging choices also shape the microbial and enzymatic load delivered to the plant: reduced condensation and optimized gas exchange can lower spoilage pressure, potentially allowing milder stabilization treatments that better retain green color and basil aroma in pesto [40,67].

Most studies focus on maintaining visual quality, chlorophyll content, and aroma in fresh leaves; however, the behavior of stored basil during grinding, emulsification, and thermal stabilization remains largely uncharacterized. In particular, the impact of storage-induced changes in enzyme activity, membrane integrity, and antioxidant capacity on pesto stability has not been systematically evaluated. This disconnect limits the ability to translate postharvest optimization into tangible improvements in product shelf-life, highlighting the need for studies that directly link storage conditions to processing outcomes.

### 4.5. Washing

Washing basil is a critical step in postharvest handling to remove surface contaminants and reduce microbial load, thereby extending shelf-life [76]. Among available methods, dipping leaves in electrolyzed oxidizing water (EOW) at concentrations of 5–20 ppm for 30 min has been shown to better preserve leaf color, maintaining higher levels of chlorophyll and carotenoids even after 14 days at room temperature [71]. The use of organic acids, such as 2% lactic acid, has proven more effective than tap water or acidic electrolyzed water (pH 2.5) in reducing populations of mesophilic bacteria as well as pathogens like *Escherichia coli* and *Salmonella typhimurium*, particularly when the solution is heated to 50 °C [77]. Combined treatments of organic acids with oxidizing agents offer additional benefits; for instance, dipping basil in a solution of 2% lactic acid and 2% hydrogen peroxide at 40 °C effectively reduced *E. coli* O157:H7 while enhancing leaf appearance and aroma after 4 days of storage at 4 °C, resulting in higher consumer acceptability [78]. Although 6% sodium hypochlorite often provides superior microbial control compared to other treatments, its use may be limited due to the formation of by-products and residual compounds [79,80]. Overall, washing basil with organic acids, optionally combined with oxidizing agents and applied at mild heat, represents a promising approach to ensure both safety and quality of fresh leaves.

From a hurdle-technology viewpoint, washing efficacy should be connected to the subsequent stabilization strategy (acidification, water activity (aw) reduction, or pasteurization), since lower initial contamination can reduce the severity of downstream hurdles and mitigate quality losses associated with heat or acidity [8,81].

### 4.6. Cold Acclimatation

Exposure to moderately cool temperatures immediately postharvest (cold acclimation) reduces chilling injury by enhancing cold-responsive gene expression, accumulating protective solutes (sugars, starch, proline), and increasing antioxidant enzyme activity. Shelf-life was extended by 5 days when harvested basil was chill-hardened 1 day prior to storage at 5 °C and compared to nonchilled controls [62].

### 4.7. Fumigation and Vapor Treatments

Nitric oxide (NO) fumigation is emerging as a postharvest strategy to reduce water loss and delay spoilage in fruits and vegetables. Although its effects have been tested on many crops, data on basil are limited. In basil, NO treatments at 1–5 μL·L^−1^ for 2 h reduced water loss by 8% after 24 h under storage conditions at 5–20 °C [82]. However, the impact of NO fumigation on basil beyond 24 h remains unknown, leaving the potential for shelf-life extension uncertain.

Vapor treatments with vinegar and tea tree oil can extend the shelf-life of fresh basil by limiting microbial growth. Fumigating basil with upland rice fermented vinegar and 4% acetic acid for 10 min increased storage in the dark at 12 °C from 17.8 to 25.4 days, whereas acetic acid alone provided a smaller improvement [83,84]. These treatments also reduce weight loss, slow chlorophyll degradation, enhance antioxidant activity, and protect cell membranes. Tea tree oil (*Melaleuca alternifolia* L.), rich in terpinen-4-ol, has antimicrobial properties and, when applied as a 1 mL·L^−1^ vapor for 1 min at 16 °C, doubled basil shelf-life from 4.7 to 10.3 days while preserving freshness, volatile oils, and enzyme activity [85,86]. Overall, vaporizing vinegar and essential oils appears to be a safe, eco-friendly method for maintaining basil quality, with limited impact on taste and aroma [83,84,87].

1-Methylcyclopropene (1-MCP) is a postharvest treatment applied to freshly harvested basil leaves or sprigs to slow senescence by blocking ethylene receptors. Single-dose applications, such as 0.4 g·m^−3^ for 8 h or 0.3 cm^3^·m^−3^ for 24 h at 15 °C, have been shown to extend shelf life by 4–6 days, reduce leaf abscission, minimize weight loss, slow chlorophyll degradation, and maintain protein and volatile oil content [74,88]. Shorter exposures or slightly different concentrations, e.g., 0.5 g·m^−3^ for 30 s, did not produce significant improvements, highlighting the importance of dosage and treatment duration.

### 4.8. Nanotechnologies

Nanoparticles have been used to prolong the shelf life of harvested basil. Thyme oil–chitosan nanoparticles increased storage at 20 °C by 2.4-fold, reducing weight loss, oxidative stress, and chlorophyll degradation while preserving volatile oils. Nano-curcumin and nano-rosmarinic acid showed similar effects during 3 weeks at 10 °C, also limiting microbial growth [89,90].

While postharvest technologies are primarily intended to preserve basil freshness and extend shelf life, they also modify key raw material attributes, including microbial load, chlorophyll retention, antioxidant status, volatile composition and tissue integrity. These changes are expected to influence processing behavior and pesto stability, although direct evidence linking postharvest treatments to product performance remains limited. Most studies focus on fresh-leaf quality rather than downstream effects on grinding, emulsification, stabilization and shelf life. Consequently, the development of integrated field-to-fork models linking postharvest management to basil technological functionality and pesto quality remains a major research challenge. The most relevant postharvest strategies and their potential technological implications for pesto production are summarized in Table 4.

## 5. Industrial Pesto Sauce Production Chain

The raw material is required to be healthy, fresh, and free from visible contamination such as soil, fertilizer or pesticide residues, and any foreign matter. The vegetative parts are to be free of flowering stems and must not exhibit abnormal odors or flavors. Leaves should be intact, displaying color and appearance consistent with the cultivar and harvest period, and free from frost damage, pest infestation, or diseases that could impair their edibility. An average stem length above 16 cm is recommended [5].

Despite the extensive characterization of basil cultivars, a critical gap remains in the standardization of raw material specifications for industrial pesto production. Studies have demonstrated significant intra-cultivar variability, particularly within “Genovese” basil, where differences in seed origin, environmental conditions, and agronomic practices can lead to substantial variation in morphological traits, essential oil composition, and phenolic content [23,26,32]. This chemotypic instability poses a major challenge for industrial processing, where consistent aromatic and functional profiles are required to ensure product standardization.

The initial stage of the industrial process involves a series of thorough washing steps aimed at ensuring the raw material is completely cleaned [91]. After the final wash, any excess water retained between the leaves must be removed using a centrifuge or another system that allows more thorough drying while minimizing damage to the product.

Damaged leaf portions and remaining foreign materials are separated as the product moves along the sorting belts. Next, the basil is ground and mixed with a combination of oil and salt, resulting in an emulsified product known as a semi-finished product [92]. This mixture is typically subjected to a heat treatment at 95 °C for 1 min to eliminate pathogenic microorganisms and ensure enzyme stabilization [40]. The semi-finished product can be stored as is or directly transformed into pesto sauce by adding other ingredients such as Grana Padano cheese, pine nuts, Pecorino cheese, salt, garlic, and occasionally additional components like citric or ascorbic acid, which act as an antioxidant and pH corrector. However, the recipes on the market show significant variability. After preparation, it undergoes stabilisation and packaging [40,93].

Due to its physicochemical properties, pesto has a naturally limited shelf-life. Therefore, to enable wide-scale distribution, industrial pesto must be processed immediately after production to extend its stability, typically through pasteurization or sterilization [93]. This requirement makes processing suitability a practical industrial constraint: variability in basil pigments, enzymes and antioxidants, shaped upstream by genotype, nutrition, light and storage, can shift the intensity of browning and oxidation during stabilization, thereby affecting the need for acidification, oxygen control, or alternative technologies [5,36].

**Figure 3:**
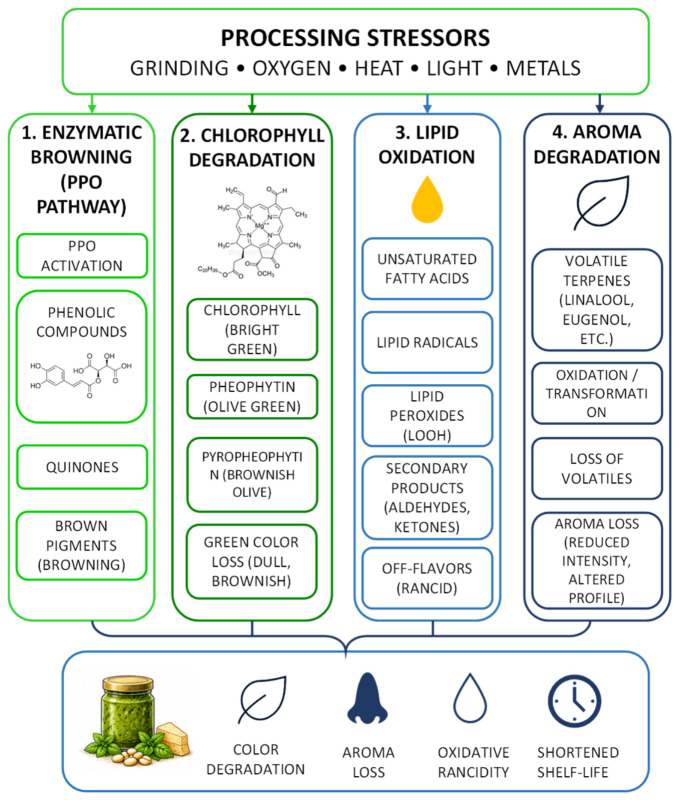
Oxidation and color degradation pathways affecting pesto quality and shelf-life. Grinding, oxygen exposure, thermal processing and storage conditions promote enzymatic browning, chlorophyll degradation, lipid oxidation and volatile loss. These interconnected mechanisms contribute to color deterioration, aroma depletion, off-flavor formation and reduced product stability.

From a physicochemical perspective, pesto can be described as a complex oil-in-water emulsion characterized by a high interfacial area between lipid and aqueous phases. This structure promotes oxidative reactions, particularly in the presence of polyunsaturated fatty acids derived from olive oil and pine nuts. Transition metals such as iron and copper can act as pro-oxidants, catalyzing radical formation and accelerating lipid degradation [33,34]. Moreover, the headspace oxygen concentration and oxygen diffusion within the emulsion matrix play a critical role in determining oxidation kinetics. Understanding the interplay between raw material antioxidant capacity and emulsion microstructure is therefore essential to predict product stability. Chlorophyll degradation during pesto processing follows well-established chemical pathways. Under acidic conditions and thermal treatment, magnesium ions in chlorophyll molecules are replaced by hydrogen, leading to the formation of pheophytins and the characteristic olive-brown color [36]. Further degradation can produce pheophorbides, intensifying color loss. The rate of these reactions is strongly influenced by pH, temperature, and oxygen exposure, suggesting that both formulation and processing conditions must be carefully controlled. Importantly, the initial chlorophyll content of basil, determined by agronomic practices, interacts with these factors, reinforcing the need for an integrated approach linking cultivation to processing stability. The major deterioration mechanisms affecting pesto quality involve interconnected pathways of enzymatic browning, pigment degradation, lipid oxidation and aroma loss. These reactions are accelerated by grinding, oxygen exposure, heat and storage conditions, ultimately determining the sensory quality and shelf-life of the final product Figure 3. From a microbiological standpoint, the stability of the product is linked to the main physicochemical parameters such as pH and water activity (aw), and possibly to heat treatment [91]. While some industries do not apply heat treatment, others prefer to ensure product stability through a pasteurization treatment, which allows storage at room temperature.

The purpose of pasteurization is to inactivate all vegetative cells; only the spores of aerobic and anaerobic microorganisms remain viable. It is therefore necessary to ensure that these spores are not able to germinate and spoil the product [81]. The most critical spore-forming pathogen in this context is Clostridium botulinum, whose germination is inhibited at pH ≤ 4.7 and aw ≤ 0.93. Pasteurization is one of the conventional food processing procedures to extend shelf-life in the food industry [64,91]. Nonetheless, this method usually results in changes in nutritional, chemical/biochemical and sensorial properties of foods that reduce their acceptance by consumers. Moreover, a huge color loss occurs. The loss of green color is mainly due to the degradation of chlorophyllic pigments. Degradation of chlorophyll a and b during thermal processing produces color changes from a bright green color to the olive brown color of pheophytins a and b [36].

Accordingly, safety-driven thermal profiles should be co-optimized with raw material functionality (chlorophyll pool, pH buffering capacity, PPO/POD activity) to minimize quality penalties, an integration rarely addressed in current basil or pesto literature [5,40].

Other preservation techniques are used as “barriers” (hurdles) to temporarily or permanently inhibit microbial growth. These include pH control, reduction in aw through the addition of salt or sugar or by drying, temperature control (freezing or refrigeration), and the addition of preservatives (such as organic acids). Acidity regulators are typically added to commercial sauces; however, the addition of these often affects the organoleptic properties of the final product [7,8].

Overall, several types of pesto can be found on the market that differ in formulation and in the preservation system applied: acidified pesto (pH < 4.6) and pasteurized pesto, pesto with aw < 0.90 and pasteurized, fresh refrigerated pesto not heat-treated, pesto not heat-treated but stabilized by a reduction in aw ≤ 0.80 capable of inhibiting microbial growth, frozen pesto, and dried pesto [91].

Although thermal treatments are currently the most widely used, non-thermal technologies are receiving increasing interest in response to consumers’ demand for minimally processed foods with fewer or no added preservatives [8]. High hydrostatic pressure (HPP), pulsed electric fields (PEF), ultrasound (US), and ultraviolet light (UV) are some of these technologies that have been increasingly studied and applied in the food industry to replace traditional processes, as they have been shown to significantly preserve the nutritional and organoleptic properties of foods while ensuring microbiological safety.

Even though non-thermal technologies such as HPP, PEF, and US have shown promise in preserving nutritional and sensory quality in plant-based foods, their application to pesto systems remains limited. HPP, for instance, has been reported to inactivate certain enzymes while preserving pigments; however, its effects on polyphenol oxidase activity, chlorophyll stability, and emulsion structure in pesto matrices are not fully understood. Additionally, pressure-induced changes in protein and lipid interactions may alter emulsion stability, potentially leading to phase separation or texture modifications. This highlights the need for product-specific validation studies rather than extrapolation from single-ingredient systems.

For pesto, these technologies must be assessed in a matrix-specific manner: enzyme inactivation, emulsion microstructure, and oxygen transfer can differ markedly from single-ingredient plant tissues, potentially changing oxidation and texture outcomes despite similar microbial lethality [7,8,34].

Despite extensive research on thermal and non-thermal technologies, their application to pesto systems remains largely empirical. Most studies extrapolate findings from single-ingredient plant matrices, neglecting the complexity of pesto as a multi-component emulsion [8,94,95]. As a result, the effects of these technologies on oxidation pathways, enzyme activity, and microstructure remain insufficiently characterized in real product conditions.

## 6. Quality Control

Beyond safety aspects, the ability to ensure consistent quality of the final product is one of the most important aspects of food industrial production. Quality controls include rigorous, multi-stage processes spanning raw materials, processing, and final product evaluation, reflecting the complexity and perishability of this oil-rich, plant-based sauce [96].

### 6.1. Raw Material

A primary control point concerns the quality of basil, the key ingredient, which is assessed in terms of cultivar-related aromatic profile and phytochemical composition. Studies have identified characteristic volatile compounds such as linalool, eucalyptol, estragole, and methyl cinnamate as markers of basil and pesto aroma quality, while antioxidant capacity and phenolic compounds (e.g., rosmarinic acid) are monitored to evaluate both sensory potential and oxidative stability. In addition, agronomic and phytosanitary aspects, including susceptibility to diseases such as downy mildew, are also considered relevant for ensuring consistent raw material quality [15,55,97,98,99].

### 6.2. Chemical and Physical Analyses

The physical properties of pesto are also routinely evaluated, as they directly influence consumer perception and product stability. Key chemical parameters such as consistency, rheological behavior, texture, pH, moisture content, and aw are commonly measured. pH tends to decrease during the initial days of storage, with final values varying according to storage temperature [96,100,101]. Macronutrient content, including proteins, lipids, and sodium, is determined using the Kjeldahl method, Soxhlet extraction, and atomic absorption spectroscopy (AAS), ensuring comprehensive characterization of the matrix [100]. Among quality attributes, color plays a particularly critical role, as a bright green appearance is strongly associated with freshness. Colorimetric analysis is generally used to monitor color, considering the parameters L*, a*, and b*, with particular focus on a*, indicative of green intensity, typical of green plant-based products [101]. Studies have shown that green intensity decreases during storage, particularly in pasteurized samples and in samples enriched with phenolic extracts; nevertheless, the lower overall color variation (ΔE) observed in enriched samples suggests that polyphenols may partially mitigate chlorophyll degradation and browning reactions [40].

### 6.3. Lipid Oxidation

From a chemical perspective, pesto is characterized by a complex matrix rich in lipids and bioactive compounds, making oxidative stability a central quality concern. Due to its emulsified nature, with extensive interface between the lipid phase and water-soluble pro-oxidants, monitoring lipid oxidation is essential. Lipid oxidation in pesto, driven by oxygen exposure, light, and heat, causes rancidity, off-flavors, and browning (pigment degradation) during storage. It involves enzymatic browning (polyphenol oxidase) and chemical oxidation of olive oil’s unsaturated fats. Routine analyses include the determination of total lipid content, phenolic composition, and antioxidant capacity, alongside the monitoring of primary and secondary lipid oxidation products. Commonly used methods include peroxide value (PV) and thiobarbituric acid reactive substances (TBARs) assays, which allow evaluation of primary peroxides and secondary lipid degradation products, respectively [33]. The composition of fatty acids, particularly the presence of polyunsaturated fatty acids (PUFAs), and the headspace oxygen concentration make pesto highly susceptible to oxidation, with increasing rancidity observed especially at elevated temperatures [34]. Research indicates that high-pressure processing (HPP) can be a better alternative to thermal processing (TP) in mitigating lipid oxidation, though it can still affect peroxide values. The analysis of browning through colorimetry confirms that controlling oxygen and using natural additives are key to maintaining the quality of the emulsion [102]. Key prevention includes antioxidants (e.g., lactoferrin) or innovative active packaging. Active biodegradable PLA packaging incorporating an oxygen scavenger effectively reduced lipid oxidation and improved quality stability in pesto, demonstrating its potential as a strategy to extend shelf-life in lipid-rich foods. The preservation of key phytonutrients, such as caffeic acid derivatives and α-tocopherol, is also considered an important quality parameter.

### 6.4. Volatile Compounds Analysis

The analysis of volatile compounds provides additional insights into both the characteristic aroma profile and the onset of quality defects. Headspace analysis techniques are used to monitor terpene composition as well as the formation of off-flavor compounds associated with lipid oxidation, thus linking chemical stability with sensory perception. Critical compounds such as diallyl disulfide and octanoic acid are correlated with garlic aroma and rancidity perception, respectively, serving as chemical markers of quality deterioration [97,99,100,103].

### 6.5. Microbiological Safety

Given that pesto is often marketed as a fresh or minimally processed product, microbiological safety is another essential aspect of quality control. Microbial analyses typically include the determination of bacterial and fungal loads at different stages of shelf-life, including after opening, in order to evaluate both product safety and secondary shelf-life behavior [100,104].

### 6.6. Sensory Evaluation

Sensory acceptability is a key component of pesto quality and depends on the perception of visual, aromatic, and textural attributes. Comprehensive sensory evaluation represents an integrative indicator of pesto quality, including attributes such as color, basil aroma, garlic, cheese, and pine nut flavor, as well as defects such as rancidity, acidity, and bitterness [103]. Assessments are performed by trained panels according to ISO protocols, using semi-quantitative scales and kinetic models to estimate shelf life based on sensory acceptability thresholds. Color is one of the most relevant quality attributes for consumers, as the characteristic bright green color of Pesto alla Genovese is associated with freshness, high-quality raw materials, and product authenticity. The preservation of green color is mainly related to chlorophyll stability, which can be affected by processing conditions, particularly pH and temperature, promoting the conversion of chlorophylls into pheophytins and resulting in undesirable browning. Instrumental color parameters (CIELab L*, a*, b*, ΔE, and browning index) are therefore widely used to monitor quality changes during processing and storage, with the a* parameter being proposed as a useful indicator of chlorophyll degradation and green color loss [105].

Aroma is another major determinant of pesto acceptability and is mainly associated with basil volatile compounds, including linalool, eugenol, estragole, and other terpenoids [100]. Their relative abundance is influenced by genotype, cultivation conditions, and harvest stage, highlighting the importance of selecting basil materials with stable aromatic profiles for pesto production [4,97,106]. Texture also contributes to consumer perception, with the ideal pesto characterized by a smooth but slightly granular consistency, resulting from the interaction among basil particles, nuts, cheese, and the oil phase. During storage, defects such as oil separation may negatively affect product appearance and acceptability, requiring strategies able to maintain structural and oxidative stability [107]. Finally, sensory evaluation remains essential for shelf-life assessment because instrumental changes do not always correspond to consumer rejection. Nicosia et al. (2021) [105] demonstrated that sensory analyses, combined with microbiological, physicochemical, and volatile compound evaluations, allowed the estimation of the secondary shelf-life of industrial pesto products under realistic domestic storage conditions, showing that consumer acceptability may be maintained beyond the labelled period.

### 6.7. Future Advancements in Pesto Quality Assessment

**Figure 4:**
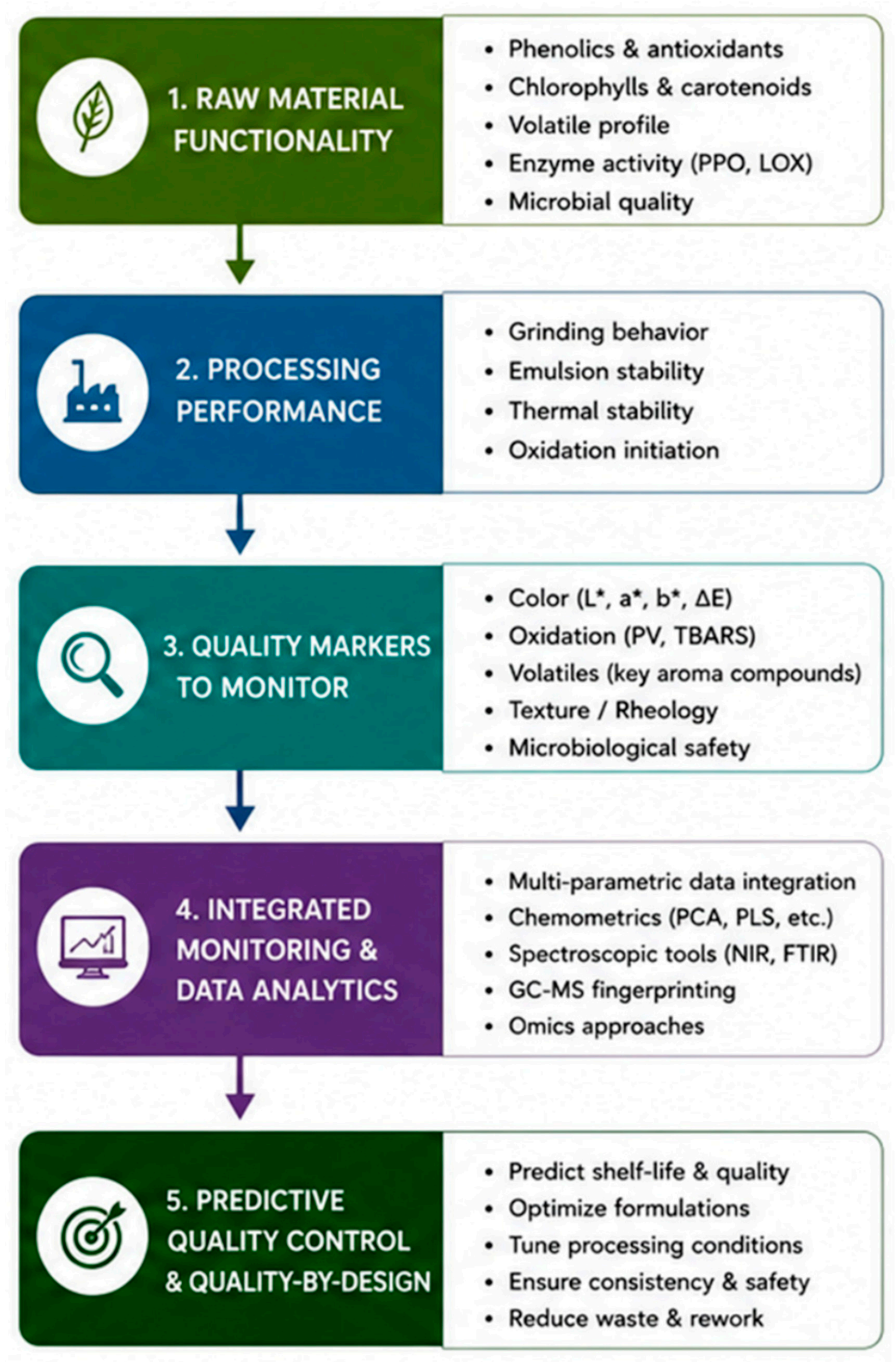
Integrated quality control and predictive framework for industrial pesto production. Raw material functionality, processing performance and quality markers are integrated through advanced analytical tools, chemometrics and predictive modelling approaches. This framework supports the development of quality-by-design strategies aimed at improving consistency, shelf-life and process optimization in industrial pesto production.

Current quality control approaches remain predominantly descriptive, focusing on isolated parameters such as color, lipid oxidation, or volatile composition. However, the lack of integration between these indicators limits their predictive power. Moving from descriptive quality assessment toward predictive quality management requires the integration of raw material composition, process parameters and product quality indicators within a unified analytical framework (Table 5). A conceptual model for this transition is proposed in Figure 4.

A transition toward multi-parametric and model-based quality control systems is therefore required, where compositional markers are directly linked to kinetic degradation processes and sensory outcomes. Ensuring consistent quality in pesto production requires the integration of advanced monitoring tools with predictive and multi-parameter analytical approaches. During processing, innovative in-line monitoring techniques have been introduced to improve process control and product standardization. In particular, near-infrared (NIR) spectroscopy combined with chemometric tools such as principal component analysis (PCA), partial least squares (PLS), and multivariate statistical process control (MSPC) charts has been successfully applied to monitor key parameters such as consistency and total lipid content in real time, as well as to detect deviations related to batch variability or processing interruptions [96]. Looking ahead, advancements in pesto quality assessment are expected to rely increasingly on predictive and multi-parametric approaches. Kinetic models based on Arrhenius equations can be applied to describe lipid oxidation, color degradation, and sensory decay, enabling the estimation of shelf-life under different storage conditions [40]. In parallel, omics-based approaches offer new opportunities for quality control and authentication. Techniques such as GC-MS fingerprinting and electronic nose systems have demonstrated potential in discriminating basil cultivars and monitoring aroma evolution [28]. Metabolomic profiling may further support traceability and verification of PDO products, linking chemical signatures to geographical origin and production practices.

While quality control systems focus on monitoring product attributes and ensuring consistency, broader considerations related to safety, regulatory compliance, and supply chain traceability are equally critical for industrial pesto production. Within industrial pesto production, safety and compliance are ensured through the implementation of structured management systems that extend beyond individual processing steps. HACCP frameworks define critical control points along the production chain, including raw material sanitation, temperature management during grinding and mixing, and control of formulation parameters such as pH, which in pesto typically remains above 5.0 and therefore does not intrinsically inhibit microbial growth [108]. In this context, safety is achieved not through a single intervention but through the combined application of preventive measures, hygienic design, and process control strategies.

In parallel, certification schemes such as Basilico Genovese PDO introduce an additional layer of standardization by imposing strict requirements on geographical origin, cultivar identity, and production practices. While primarily aimed at preserving authenticity and typicity, these schemes also influence raw material consistency and processing behavior, indirectly affecting industrial performance and quality reproducibility.

At the supply chain level, traceability systems play a key role in linking primary production to processing and distribution. However, despite the increasing availability of digital tools, including blockchain-based platforms and sensor-integrated monitoring systems, their application in pesto production remains limited.

Current approaches are largely based on batch-level traceability, with limited integration of continuous process data, thereby constraining the development of predictive, data-driven quality and safety management systems.

Beyond safety and regulatory compliance, the sustainability of basil cultivation and pesto production is emerging as a critical dimension influencing both industrial practices and consumer perception [109]. Without the development of integrated, data-driven approaches linking raw material variability, processing kinetics, and quality outcomes, advancements in pesto production will remain incremental rather than transformative.

## 7. Conclusions

This review highlights that the quality of industrial pesto cannot be fully understood through isolated analyses of agronomic practices, postharvest handling, or processing technologies. A field-to-fork perspective is instead required, considering basil a dynamic raw material whose functionality evolves across the entire value chain.

The concept of “raw material functionality” links pre-harvest factors (e.g., genotype, environmental conditions, and crop management) to technological performance during processing and storage. Agronomic interventions can significantly modulate basil phytochemical composition (including phenolics, volatile compounds, and chlorophyll content), yet these changes do not always translate into predictable improvements in pesto stability, due to the complex interactions occurring in emulsified, oxygen-sensitive systems.

Multiple trade-offs across the value chain: strategies that enhance functional quality often reduce yield or increase variability, while safety-driven interventions may compromise color and sensory quality. Integrated optimization approaches that simultaneously consider agronomic, technological and sensory dimensions are therefore needed.

Despite significant advances, several critical gaps remain. In particular, predictive models linking raw material composition to shelf-life performance are still lacking, postharvest and processing studies are rarely connected, and the behavior of basil under real processing conditions (e.g., grinding, emulsification, thermal stabilization) remains insufficiently characterized.

Non-thermal technologies and advanced packaging solutions also require further validation in pesto-specific matrices.

Future research should therefore move beyond descriptive approaches and develop multi-parametric predictive frameworks that integrate compositional markers of basil (e.g., phenolics, volatiles, pigments) with kinetic indicators of degradation (e.g., lipid oxidation, color change, sensory decay). Particular attention should be paid to linking postharvest handling directly to processing behavior and final product stability, while validating non-thermal technologies and innovative packaging solutions under real pesto matrix conditions. The integration of chemometrics, omics approaches and real-time monitoring tools will be essential to support this transition toward quality-by-design strategies.

Ultimately, improving industrial pesto quality will depend on the convergence of agronomy, food chemistry, process technology, microbiology, and sensory science. Establishing this interdisciplinary dialogue will enable the design of basil cultivation and processing strategies optimized not only for yield and composition, but for technological functionality and long-term product stability. Without this integration, efforts to improve pesto quality will remain incremental rather than transformative.

## Figures and Tables

**Table 1:** Fresh-market quality versus industrial processing suitability of sweet basil.

Fresh-Market Quality Attribute	Industrial Functionality
Appearance	Color retention
Fresh aroma	Aroma persistence
Tenderness	Processability
Freshness	Storage stability
Consumer appeal	Technological performance

**Table 2:** Functional attributes defining basil raw material functionality.

Functional Attribute	Measurable Indicators	Industrial Relevance
Color functionality	chlorophyll, carotenoids, ΔE (Color retention)	green color
Aroma functionality	linalool, eugenol, volatile fingerprint	aroma retention
Oxidative functionality	phenolics, antioxidant capacity, PV (Peroxide value)	shelf-life
Enzymatic functionality	PPO (polyphenol oxidase), POD (peroxidase)	browning
Structural functionality	water status, tissue integrity	grinding
Rheological functionality	emulsion viscosity	texture
Microbiological functionality	microbial load	safety

**Table 3:** Agronomic interventions affecting basil raw material functionality and their expected implications for industrial pesto production.

Agronomic Factor	Quantitative Effect on Basil	Potential Implications for Pesto Functionality	Reference
Successive harvests (2nd cut vs. 1st cut)	Fresh biomass increased by 172% and total phenolic acids by 413%. Eugenol increased by 75.3% and trans-α-bergamotene by 48.2% in cv. Eleonora.	Higher antioxidant potential and aroma intensity; possible improvement in oxidative stability and sensory persistence.	[4]
Hydroponic cultivation (vs. soil cultivation)	Pesto produced from hydroponic basil showed higher chlorophyll (18.7 vs. 15.6 mg kg^−1^), carotenoids (132 vs. 112 mg kg^−1^), total phenolics (176 vs. 159 mg 100 g^−1^), and antioxidant activity (276 vs. 242 mg TEAC kg^−1^). Pesto from hydroponic basil exhibited higher viscosity (15,656 vs. 13,814 mPa·s) and shear stress (695 vs. 681 Pa) and better sensory acceptance.	Improved color retention, oxidative stability, rheological properties, sensory quality, and microbial shelf-life of pesto.	[13]
Moderate water deficit (75–50% Field Capacity)	Reduction in aroma-related volatiles including linalool, nerol, geraniol and eugenol; losses during storage reached approximately 50% for several volatiles in some genotypes.	Potential reduction in fresh basil aroma and lower volatile retention in pesto during storage.	[35]
Deficit irrigation (50% Field Capacity)	Essential oil concentration increased to 1.10%, while green herb yield decreased from 2269 to 908 kg ha^−1^-equivalent (kg da^−1^ values reported) and drug leaf yield from 301 to 131 kg da^−1^	Higher aroma concentration but lower raw material productivity.	[41]
Mild water stress (60% Field Capacity)	Eugenol increased from 16.2% to 50.8% during the vegetative stage, while linalool proportion decreased.	Shift in aroma balance, potentially affecting pesto sensory profile and increasing batch-to-batch variability	[45]
High irradiance (24.9 vs. 5.3 mol m^−2^ d^−1^)	Total volatile oil content increased; linalool and eugenol increased under high irradiance, while methyl eugenol increased under low irradiance. Heavy shading (75%) strongly reduced plant biomass and leaf area.	Improved characteristic Genovese aroma profile and greater flavor intensity in pesto.	[46]
Moderate pre-harvest UV-B (1 h d^−1^ for 2 d)	Phenolics increased by 28–126%, flavonoids by 80–169%, anthocyanins by 9–18%; however high UV-B doses reduced fresh biomass by 12–51% in green basil.	Enhanced antioxidant content, but excessive UV-B may compromise industrial yield.	[39]
AMF (arbuscular mycorrhizal fungi) *Glomus intraradices* + water stress	Increased plant height and partially compensated stress effects on growth No significant deterioration of EO profile compared with stressed plants without AMF mitigation	Increased plant height and partially compensated stress effects on growth Potential strategy to improve sustainability while preserving aromatic quality	[45]
High potassium nutrition (5.0 vs. 1.0 mM K)	Total phenolics significantly increased; rosmarinic acid and chicoric acid concentrations increased; antioxidant capacity (DPPH and FRAP) significantly enhanced at 5.0 mM K.	Greater antioxidant potential and improved oxidative stability of pesto.	[37]
Potassium enrichment (13 vs. 7 mmol L^−1^ K)	Total phenolics increased from 12.3–13.5 to 14.8 mg GAE g^−1^ extract; total flavonoids increased from 5.38 to 6.64 mg QE g^−1^ extract.	Increased antioxidant reservoir and improved nutraceutical quality of basil destined for pesto processing.	[50]
Moderate phosphorus supply + AM fungi under drought	Mycorrhizal plants at the intermediate P level (P2) showed increases of 1.84-fold (TPC), 1.59-fold (rosmarinic acid) and 2.22-fold (caffeic acid) compared with non-mycorrhizal plants.	Enhanced antioxidant composition and preservation potential of pesto.	[51]
Beneficial microbial consortium (Trichoderma + Azotobacter)	Rosmarinic acid content increased by 110%; total fresh biomass increased by 23.6% with BP + T22 + 76A and by 26.3% with BP + 6PP.	Simultaneous improvement of basil yield and antioxidant quality, supporting higher functional quality of pesto.	[59]
PGP bacteria associated with AMF spores	Expression of key genes involved in rosmarinic acid biosynthesis increased: TAT + 5.7-fold, HPPR + 2-fold, CS3’H iso1 + 2.4-fold.	Potential stimulation of rosmarinic acid biosynthesis and enhancement of antioxidant functionality.	[58]
Nitrate reduction through NO_3_^−^/Cl^−^ management	Leaf nitrate decreased from 32.1 to 1.1 g kg−1 DW when NO_3_^−^:Cl^−^ ratio changed from 80:20 to 20:80; however shoot biomass decreased by 38% (113.2 to 70.1 g plant^−1^).	Lower nitrate accumulation but reduced yield; possible enhancement of functional quality at the expense of productivity.	[52]
Moderate salinity (20 mM NaCl)	Leaf area decreased by 18%, fresh yield by 17%, and shoot dry weight by 19%, while antioxidant activity and phenolic accumulation increased.	Potential improvement of oxidative stability due to enhanced antioxidant pool, although with lower biomass availability.	[54]
Higher salinity (40 mM NaCl)	Fresh yield decreased by approximately 31% and leaf area by approximately 32%; root-to-shoot ratio increased by 46%.	Increased phytochemical concentration but potentially lower industrial productivity and greater raw-material variability.	
Selenium biofortification (12 mg Se L^−1^)	Total phenols increased from 15.0 to 31.5 mg GAE g^−1^ DW (+110%), rosmarinic acid from 25.5 to 50.1 mg g^−1^ DW (+96%), and antioxidant capacity from 242 to 380 µmol Fe(II) mg^−1^ DW (+57%).	Greater antioxidant potential and expected improvement of oxidative stability in pesto.	[55]
Selenium biofortification (4 mg Se L^−1^)	Reduced ethylene production after storage and improved shelf-life preservation without yield penalties.	Potential extension of basil shelf-life before processing and improved preservation of quality attributes.	
Successive harvests (2nd cut vs. 1st cut)	Fresh biomass increased by 172% and total phenolic acids by 413%. Eugenol increased by 75.3% and trans-α-bergamotene by 48.2% in cv. Eleonora.	Higher antioxidant potential and aroma intensity; possible improvement in oxidative stability and sensory persistence.	[4]

**Table 4:** Postharvest treatments affecting basil quality and their potential technological implications for pesto production.

Postharvest Treatment	Quantitative Effect on Basil	Potential Technological Implications for Pesto Production	Reference
Controlled atmosphere (1.5% O_2_, 0% CO_2_)	Shelf life extended from 18 to 45 d in micro-perforated bags.	Improved retention of volatile compounds, chlorophyll and antioxidants prior to processing.	[1]
Moderate refrigeration	Shelf life maximized at 12–15 °C; storage life decreased from 12.5 d at 15 °C to 3.2 d at 5 °C and 1.6 d at 0 °C. Chilling injury appeared after 3 d at 5 °C and 1 d at 0 °C.	Better preservation of aroma, chlorophylls and antioxidant compounds before processing; reduced enzymatic browning risk during grinding.	[62]
Cold acclimation (10 °C preconditioning)	Chill hardening at 10 °C for 4 h prior to storage at 5 °C increased average shelf life from 4.5 to 8.2 d (+82%).	Greater preservation of leaf integrity and reduced chilling-induced quality losses before pesto manufacture.	
Electrolyzed oxidizing water (EOW)	Immersion in 5–20 ppm EOW for 30 min preserved chlorophyll and carotenoids after 14 d at room temperature.	Better green color retention in basil and potentially reduced color degradation in pesto.	[6,71]
Lactic acid washing (2%) + mild heat (50 °C)	Mesophilic bacteria reduced by 4.62 log CFU g^−1^, *Salmonella typhimurium* by 3.80 log CFU g^−1^, and *E. coli* by 3.61 log CFU g^−1^.	Lower microbial load entering processing, enabling milder stabilization strategies and potentially improving sensory quality retention.	[77]
2% lactic acid + 2% H_2_O_2_ at 40 °C	Lowest *E. coli* O157:H7 population (2.35 log CFU g^−1^) and maintenance of acceptable sensory quality for 48 h at 4 °C.	Improved hygienic quality while preserving appearance and aroma of basil used for pesto.	[78]
Nitric oxide fumigation (1–5 μL L^−1^, 2 h)	Reduced postharvest water loss by approximately 20% across horticultural commodities; basil included among tested species.	Better preservation of turgor and freshness, potentially improving grinding behavior and emulsion formation.	[82]
Vinegar vapor (4% acetic acid, 10 min)	Shelf life increased from 19.6 to 26.4 d (+35%); fresh weight loss reduced from 21.1% to 8.8% after 25 d. Chlorophyll retention and antioxidant activity improved.	Better preservation of color, antioxidant status and membrane integrity before pesto production.	[84]
1-Methylcyclopropene (1-MCP)	Applications of 0.4 g m^−3^ for 8 h or 0.3 cm^3^ m^−3^ for 24 h extended shelf life by 4–6 d, reduced chlorophyll degradation, leaf abscission and weight loss.	Improved retention of green color and volatile compounds, supporting higher pesto quality consistency.	[88]

**Table 5:** Main quality attributes of raw basil (cv Basilico Genovese DOP as gold standard) and of the final pesto, grouped by matrix and by type of quality characteristic.

Matrix	Quality Characteristic (Type)	Description/Value	Source
Basil (raw material) cv *Genovese* as gold standard, if available data	Morphological/appearance	Leaves: elliptical (oval) blade, flat or slightly convex	[21]
		Margins slightly dentate; surface minimally or not at all blistered (bollosità)	
		Color: bright, vivid green (not dark); tender, concave young leaves	[21,98]
		Plant: medium-high to tall habit, expanded or cylindrical canopy	[21]
		No mint/anise (mentholated) note—undesirable for pesto	
		Defect to avoid: leaf yellowing/browning (downy mildew) impairs marketability	[15,31]
	Aromatic/volatile	Linalool (key aroma marker): 54.57–60.08% of essential oil (cv *Genovese*)	[26]
		Eugenol: 16.21–17.93% of essential oil (cv *Genovese*)	
		Estragole (methyl chavicol): absent—very low (0.37% of essential oil)—required by the PDO specification	[26,99]
		Eucalyptol: 7.31–8.46% of essential oil (cv *Genovese*)	[26]
	Chemical–compositional (phenolics)	Caffeic acid: 16.10–50.67 (μg g^−1^ dw)	[30]
		Chicoric Acid: 32.74–205.20 (μg g^−1^ dw)	
		Rosmarinic Acid: 14.71–326.03 (μg g^−1^ dw)	
		Ferulic Acid: 2.96–12.07 (μg g^−1^ dw)	
		Total Polyphenols: 66.50–587 (μg g^−1^ dw)	
		Total phenolic acids: 766.77–1080.79 mg kg^−1^ dw	[4]
		Nitrate: 473.78–1616.41 mg kg^−1^ fw	
	Color/pigments	SPAD Index: 30.63–32.54	[4]
		Leaf color: L*: 44.69–46.46 a*: −6.76–−11.27 b*: 14.94–29.90	[4,30]
Final pesto (processed product)	Visual appearance (how the pesto should look)	Color: bright, vivid green (‘brilliant’ green); browning/muddy-green indicates oxidation and is a defect	[40,98]
		Texture: oil-in-water emulsion	[40]
		Aroma: retains characteristic basil-derived sensory notes; free of rancid/oxidised notes	[35,106]
	Color/pigments (instrumental)	Chlorophyll: 18.7 (hydroponic) vs. 15.6 mg kg^−1^ (soil basil pesto)	[13]
		Carotenoids: 132 (hydroponic) vs. 112 mg kg^−1^ (soil)	
		Color degradation rate increased with temperature (0.025 at 4 °C to 0.045 at 12 °C).	[40]
		L*, a*, b*, ΔE: CIELab tracked L* increased, whereas a* and b* decreased with increasing storage time	[93]
		Non-pasteurized pesto sauce L: 42.09 a*: −5.45 b*: 14.98 Pasteurized pesto sauce L: 41.42 a*: −1.65 b*: 14.97	[93]
	Nutritional/antioxidant	Total phenolics: 176 (hydroponic) vs. 159 mg 100 g^−1^ (soil)	[13]
		Antioxidant activity: 276 (hydroponic) vs. 242 mg TEAC kg^−1^ (soil)	
	Rheological/physical	Viscosity: 15,656 (hydroponic) vs. 13,814 mPa·s (soil basil pesto)	[13]
		Shear stress: 695 (hydroponic) vs. 681 Pa (soil)	
		Torque: 3.1 mN·m (hydroponic) vs. 2.8 (soil)	
	Physico-chemical	Acidified: pH < 4.6 + pasteurization low aw. aw < 0.90 + pasteurization Fresh refrigerated: cold storage only Aw-stabilized, reduced aw without heat treatment	[40]
		Peroxide value (PV) & TBA: apparent first-order increase during storage	[40]
	Microbiological shelf life	45 ± 2.8 days (4 °C), 30 ± 2.4 days (8 °C), 26 ± 2.1 days (12 °C).	[40]
	Sensory	Limiting shelf-life criterion: sensory (not microbial); overall acceptability ∝ peroxide value	[40]

## Data Availability

No new data were created or analyzed in this study. Data sharing is not applicable to this article.
